# Activity of Spray-dried Microparticles Containing Pomegranate Peel Extract against *Candida albicans*

**DOI:** 10.3390/molecules170910094

**Published:** 2012-08-24

**Authors:** Eliana Harue Endo, Tânia Ueda-Nakamura, Celso Vataru Nakamura, Benedito Prado Dias Filho

**Affiliations:** 1 Post Graduate Program in Pharmaceutical Sciences, Universidade Estadual de Maringá, Av. Colombo, 5790, Maringá 87020-900, Parana, Brazil; Email: eliharue@hotmail.com; 2 Department of Health Sciences, Universidade Estadual de Maringá, Av. Colombo, 5790, Maringá 87020-900, Parana, Brazil; Email: tunakamura@uem.br (T.U.-N.); cvnakamura@uem.br (C.V.N.)

**Keywords:** microparticles, pomegranate, antifungal activity, *Candida albicans*

## Abstract

Pomegranate has attracted interest from researchers because of its chemical composition and biological properties. It possesses strong antioxidant activity, with potential health benefits, and also antimicrobial properties. The aim of this study was to produce microparticles containing pomegranate extract by the spray-drying technique, utilizing alginate or chitosan as encapsulating agents. Characterization and antifungal assays were carried out. Production yields were about 40% for alginate microparticles and 41% for chitosan. Mean diameters were 2.45 µm and 2.80 µm, and encapsulation efficiencies were 81.9% and 74.7% for alginate and chitosan microparticles, respectively. The spray-drying process preserved the antifungal activity against *Candida albicans*. These results could be useful for developing dosage forms for treating candidiasis, and should be further investigated in *in vivo* models.

## 1. Introduction

The pomegranate *Punica granatum* (family Punicaceae) is a small tree originating from Asia and cultivated throughout the Mediterranean region, China, India, South Africa, and the Americas. Since ancient times, it has been used to treat several diseases. In recent times, the plant has attracted the interest of researchers in examining its composition and biological properties. The fruit is consumed mainly fresh or in beverages, and is a rich source of phenolic compounds, including hydrolyzable tannins, which possess high antioxidant activity. Ellagitannins are the major polyphenols found in pomegranate fruit [1,2,3]. Pomegranate has potential for the prevention and treatment of inflammation and cancer, and shows an antiproliferative effect against human oral, colon, prostate and breast-cancer cell lines, inhibition of gastric ulceration, and an effect against oxidative damage in diabetic rats [1,2,3,4,5,6]. Concerning its antimicrobial properties, pomegranate inhibits the growth of *Staphylococcus aureus*, methicillin-resistant *Staphylococcus aureus* (MRSA), *Listeria monocitogenes*, *Yersinia enterocolitica*, *Escherichia coli*, human influenza virus H3N2, and *Candida albicans*. Furthermore, it shows a synergistic effect against *S. aureus* when combined with antibiotics, against H3N2 when combined with oseltamivir, and against *C. albicans* when combined with fluconazole [7,8,9,10,11,12]. Punicalagin, the main ellagitannin in pomegranate, has low cytotoxicity *in vitro* and is not toxic to rats when administered orally. A pomegranate ellagitannin-enriched dietary supplement proved to be safe for humans after 28 days of treatment, and no changes have been reported in hematological, biochemical, or urinary analyses [13,14,15].

*Candida* species are typically harmless saprophyte yeasts, a normal component of the human biota in the gastrointestinal tract and oral and vaginal mucosae. These yeasts can cause superficial infections such as thrush and vaginitis; however, if the immune defenses of the host become compromised, they can cause severe systemic infections. Risk factors for patients include infection by the human immunodeficiency virus (HIV), anticancer therapy, organ transplantation, abdominal surgery, catheters, diabetes, and the use of broad-spectrum antibiotics [16,17]. The prevalence of opportunistic oral fungal infections has increased. *Candida* spp. are a potential causative agent in denture-induced stomatitis. Yeast cells adhere to and colonize oral surfaces including mucosa and irregularities in acrylic dentures, and have the ability to co-aggregate with oral bacteria. *Candida*-associated denture stomatitis is a common recurring disease observed in otherwise healthy denture wearers. The etiology of the disease is multifactorial, and systemic factors associated with continuous denture wearing, denture cleanliness, denture base material, and age of the denture are probable causes of this infection. Denture stomatitis is characterized by inflamed mucosa, a burning sensation, discomfort, or bad taste [18].

Spray drying is an important and widely applied technique in the pharmaceutical field. It is a feasible method for preparing microparticles, involving a one-step process, and is an alternative to microencapsulation methods that utilize different steps and organic solvents. Atomization occurs by compressed air, which breaks the liquid fed to the nozzle into small droplets, and the solvent in these droplets is quickly evaporated by high temperature. It is applicable to heat-resistant and heat-sensitive drugs, water-soluble and insoluble drugs, and hydrophilic and hydrophobic polymers. Furthermore, it can be adapted to industrial-scale production [19,20]. The administration of drugs loaded in microparticles benefits from both protection of the encapsulated drug, and controlled release to achieve the therapeutic effect. A desirable result is high encapsulation efficiency and preservation of the drug activity during the process of encapsulation and storage [21].

Using natural biopolymers in drug delivery is interesting because of their biodegradability and biocompatibility. Chitosan is a natural cationic polysaccharide obtained from *N*-deacetylation of chitin, and alginate is a natural anionic polysaccharide of the linear copolymers α-L-guluronate and β-D-mannuronate. Both are suitable for use in biomedical applications. Therapeutic agents such as anticancer and anti-inflammatory agents, antibiotics, and proteins have been incorporated into chitosan microparticles. Sinha *et al.* [22] reviewed the methods for preparation and agents that had been incorporated in chitosan microparticles to that date. Alginate microparticles were developed for enzyme administration, and protective agents prevented enzyme inactivation [23].

In this study, chitosan and alginate microparticles containing pomegranate extract were produced by the spray-drying technique, and they were characterized *in vitro* and their antifungal activity was assayed and compared to the extract (powder).

## 2. Results and Discussion

### 2.1. Microparticle Preparation

Spray drying is an attractive technique for microparticle preparation, because it is a rapid particle-formation process that involves only the preparation of a solution containing the drug and polymer. The process results in a homogeneous distribution of the drug and polymer. The yields for spray-dried microparticles were relatively low, about 40% for alginate microparticles and 41% for chitosan (Table 1). Low yields are frequent in the spray-drying method, which could be attributed to the small amount of materials processed (15 g) and to the loss of the smallest and lightest particles through the atomization process [20].

**Table 1:** Composition and production yields of spray-dried microparticles.

Microparticle composition	Extract:polymer ratio	Theoretical extract content (%)	Actual extract content (%)	Encapsulation efficiency (%)	Production yield (%)	Mean diameter (µm)
Alginate	1:2	33.3	27.3 ± 2.2	81.9 ± 6.5	40 ± 1.2	2.45 ± 1.37
Chitosan	1:2	33.3	24.9 ± 1.4	74.7 ± 7.2	41 ± 1.0	2.80 ± 1.28

### 2.2. Scanning Electron Microscopy

**Figure 1:**
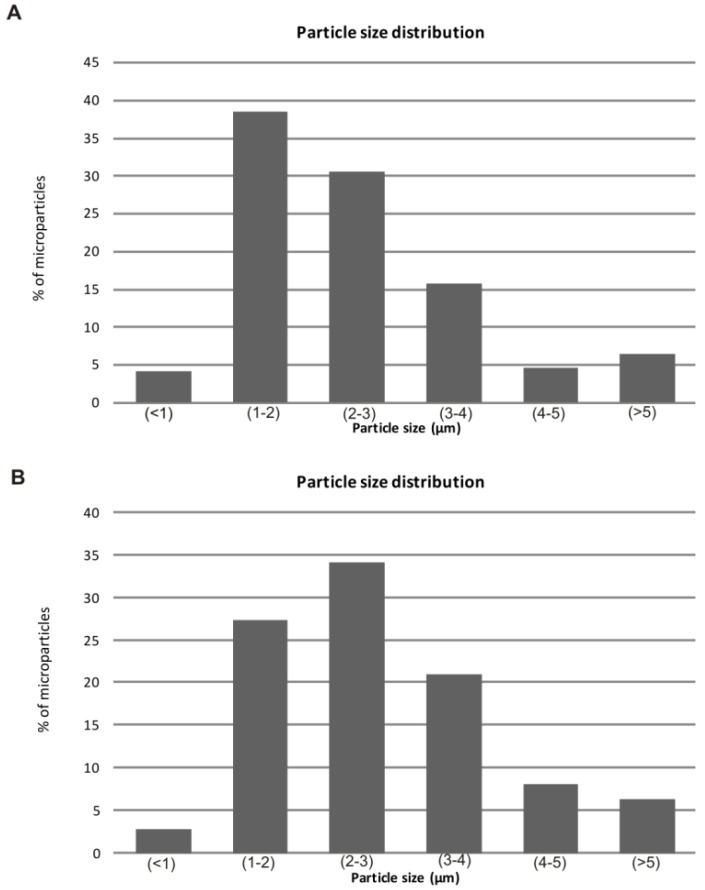
Extract loaded-microparticle size frequency distribution. (**A**) Alginate microparticle; (**B**) Chitosan microparticle.

**Figure 2:**
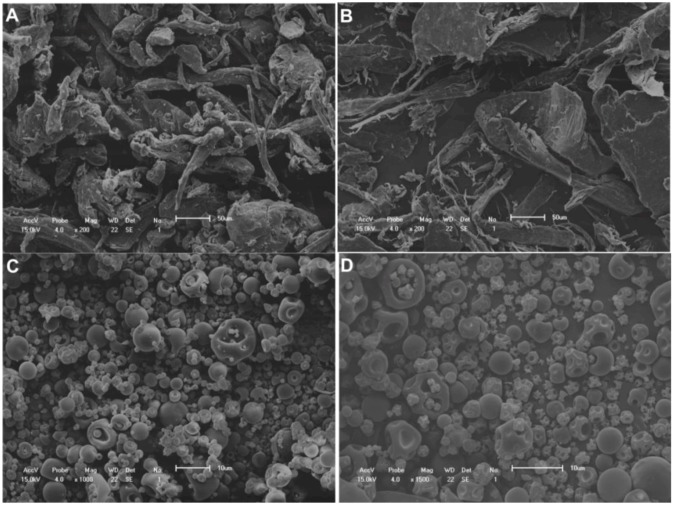
SEM images. (**A**) Alginate powder; (**B**) Chitosan powder; (**C**) Spray-dried alginate microparticles containing pomegranate extract; (**D**) Spray-dried chitosan microparticles containing pomegranate extract. Size bars: 50 µm in figures (**A**) and (**B**); 10 µm in figures (**C**) and (**D**).

Both the alginate and chitosan microparticles were small, with a spherical shape and a smooth surface. The size analysis showed that the alginate microparticles had a mean diameter of 2.45 µm, and the chitosan microparticles had a mean diameter of 2.80 µm. Figure 1 shows the microparticle size-frequency distribution. SEM micrographs also revealed morphological changes in the encapsulating agents after the spray-drying process, and the absence of extract crystals and aggregates of microparticles (Figure 2).

### 2.3. Determination of Extract Content on Microparticles

**Figure 3:**
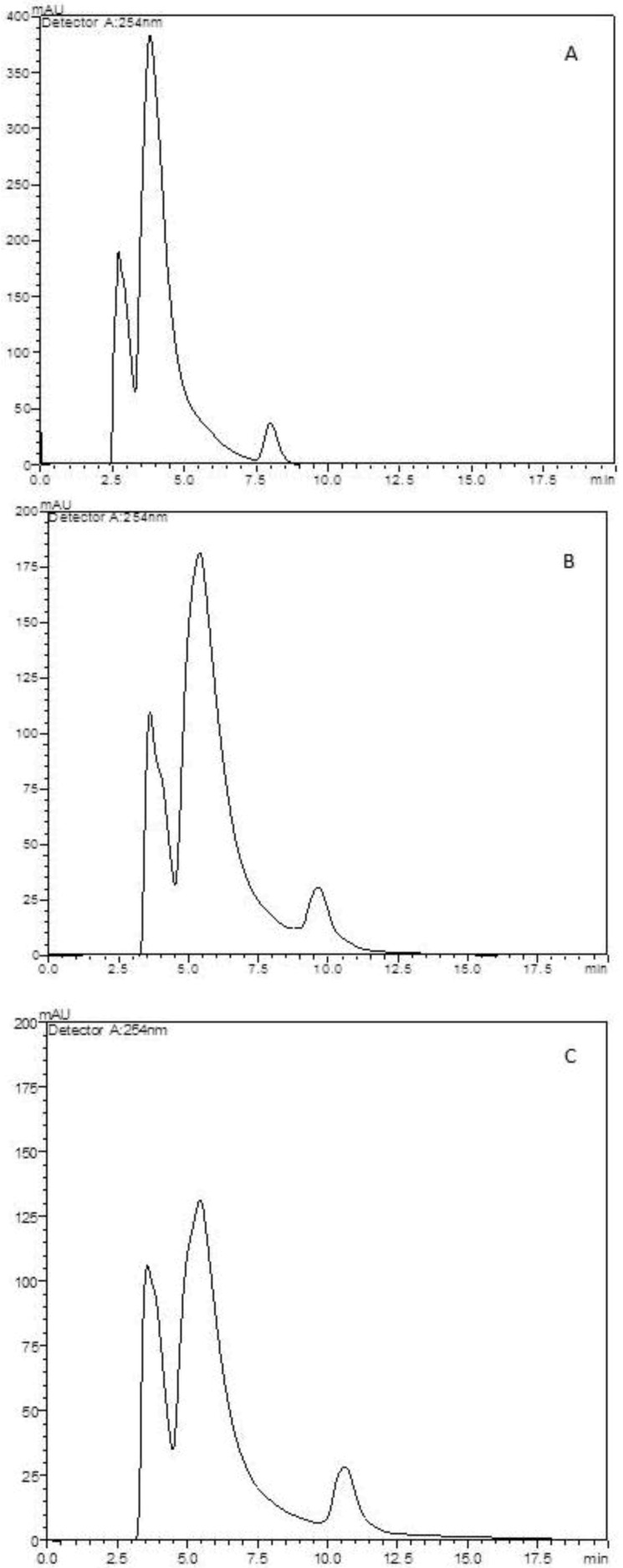
HPLC chromatograms. (**A**) Pomegranate extract solution chromatogram; (**B**) Extract-loaded microparticle chromatogram; (**C**) Extract-loaded chitosan microparticles. Run time 20 min, flow rate 1.0 mL/min and wavelength 254 nm.

The encapsulation efficiency was determined by the ratio between the actual extract content and the theoretical content. Assuming that all the extract present in the solution which was atomized was entrapped in microparticles, the theoretical extract content was 33.3% (w/w) for both types of microparticles. Reversed-phase HPLC analysis showed that the actual extract content in the alginate microparticles was 27.3%, which corresponds to an encapsulation efficiency of 81.9%. For the chitosan microparticles, the actual extract content was 24.9% and the encapsulation efficiency was 74.7% (Table 1). A calibration curve was done previously to quantify extract content. Chromatograms are shown in Figure 3.

### 2.4. *In vitro* Extract Release Test

**Figure 4:**
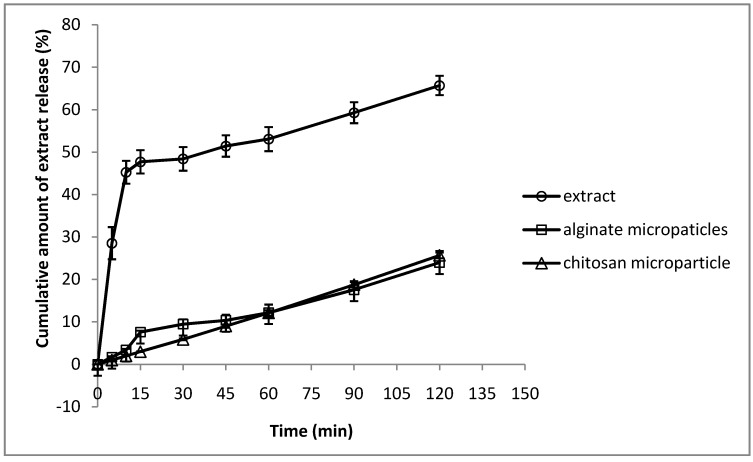
*In* *vitro* release behavior of extract (powder), extract from alginate microparticles and extract from chitosan microparticles.

Figure 4 shows the *in vitro* release behavior of the extract-loaded microparticles compared to the dissolution profile of the extract alone. As the figure shows, 65.7% of the extract was dissolved after 120 min, while the alginate and chitosan microparticles only released 23.9% and 25.6%, respectively during the same time period. The extract alone dissolved more rapidly than it was released from the microparticles. This prolonged period of release from microparticles could be useful in treatment of oral candidiasis, making repeated administration of the drug unnecessary. Taking into account the extract content and the quantity released from the microparticles, it would be possible to maintain a suitable concentration of extract capable of inhibiting the growth of *C. albicans*.

### 2.5. Antifungal *in Vitro* Assay

For *Candida tropicalis* ATCC 28707, *Candida parapsilosis* ATCC 22019 and *C. albicans* ATCC 10231 MIC (MFC) values for extract were 3.9 (3.9) µg/mL, 3.9 (>250) µg/mL and 3.9 (>250) µg/mL, respectively. For extract and extract-loaded microparticles MIC values against *C. albicans* were 3.9 and 15.6 µg/mL respectively, and were the same for both the alginate and chitosan microparticles. Extract-empty microparticles were also tested but did not show an antifungal effect. Microparticles were not tested against *C. tropicalis* or *C. glabrata*. Extract-loaded microparticles showed higher MIC values than did the extract alone. However, considering the actual extract content of 27.3% and 24.9%, corresponding to 4.2 and 3.8 µg/mL of extract, this means that the antifungal activity of the extract in the loaded-microparticle was maintained. Bruschi *et al.* [24] reported that microencapsulation of propolis by the spray-drying technique preserved its activity against *S. aureus*. Giunchedi *et al.* [20] reported an improvement of the anti-candidal activity of spray-dried chitosan microparticles containing chlorhexidine. Previous studies showed isolation and identification of punicalagin from pomegranate extract, which had inhibitory effect on *C.albicans* growth and synergic effect when associated to Fluconazole. Reduction on *C. albicans* adherence on cover glasses and morphologic alterations were caused by treatment with punicalagin [10].

### 2.6. *In Vitro* Biofilm Assay

Crystal violet (CV) staining and a MTT reduction assay were used to assess the effect of pomegranate extract, fluconazole and nystatin on pre-formed biofilm of *C. albicans*. Table 2 shows MIC values for planktonic cells and biofilm. The inhibitory effect of extract and antifungals were measured as absorbance of treated cells relative to those untreated cells (considered to be 100%). CV_50_ and MTT_50_ represent concentrations which cause 50% of reduction of absorbance observed by Crystal Violet and MTT assays, respectively. Extract and antifungal agents were less active against biofilm than planktonic cells. For extract and fluconazole, CV_50_ values were about 16 and 32 times higher than corresponding values for planktonic cells, MTT values were at least 256 and 128 times higher, respectively. For nystatin, CV_50_ and MTT_50_ were about 2 and 10 times higher than values for planktonic cells, respectively. Other studies also indicate that *C. albicans* biofilms are resistant to antifungal agents and plants extracts [25,26]. These results could be explained by differences between CV and MTT assay. CV stains all the cells in biofilm, viable or not, in contrast, MTT is reduced by mitochondrial enzymes of viable cells with a linear relation between MTT reduction and metabolic activity. These obtained results may be related to the high ability of this specie to form hyphae that may be inhibited in cells treated, reducing the total biofilm biomass, when compared with non-treated cells (control). Also, there are multiple physical, chemical, and biological factors that could influence the binding of CV to biofilms. These factors may include structural factors that affect dye diffusion, as well as morphological and physiological differences in individual cells that influence dye binding or chemical interactions between plants extract components and CV. On the other hand, there are some with the MTT reduction assay. It is well known that cells in biofilms display properties dramatically different from those of their free-living counterparts. These surface-associated changes may include alterations in MTT metabolism by biofilm cells. Others studies showed some differences between CV and MTT results. However, MTT could be a good approach to test drug susceptibility on biofilms once it indicates viable cells [27,28].

**Table 2:** MICs of pomegranate extract and antifungal agents against planktonic and biofilm of *Candida albicans* ATCC 10231.

	MIC (µg/mL)		
	Planktonic cells	Biofilm at 48 h	
		CV_50_	MTT_50_
Pomegranate Extract	3.9	62.5	>1000
Fluconazole	7.8	250	>1000
Nystatin	3.1	7.8	31.2

### 2.7. Cytotoxicity

**Figure 5:**
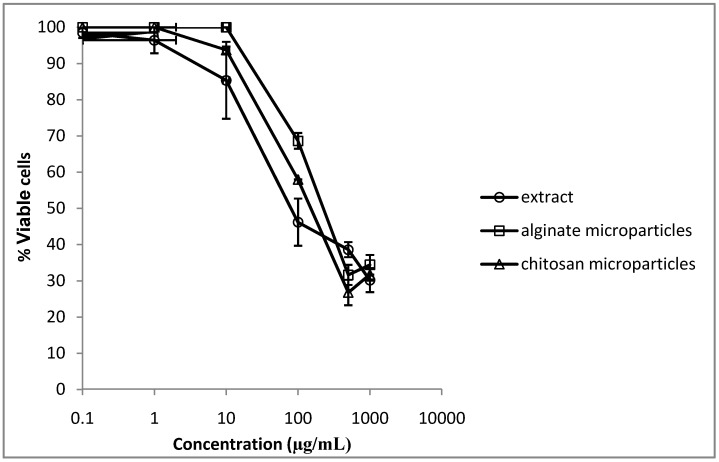
Cytotoxicity assay. Percentage of viable cells after 48 h of incubation with pomegranate extract, pomegranate extract-loaded alginate microparticles and pomegranate extract-loaded chitosan microparticles.

Vero cells were used to evaluate the potential cytotoxic effects of the pomegranate extract and the extract loaded-microparticles. After 48 h of incubation, the CC_50_ of the extract was 80 µg/mL for Vero cells. This concentration is 20 times the minimum necessary to inhibit *C. albicans* growth. For the alginate and chitosan extract-loaded microparticles, these values were 224 µg/mL and 150 µg/mL, respectively (Figure 5).

## 3. Experimental

### 3.1. Extract Preparation

*P. granatum* peels were cut into small pieces and extracted during seven days in 50% ethanol (v/v) at room temperature. The resulting yellowish extract was filtered, concentrated and lyophilized, yielding the peel crude extract [10], which was microencapsulated.

### 3.2. Alginate Microparticles Preparation

Both alginate and chitosan microparticles were obtained by spray-drying technique. First, alginate (Sigma-Aldrich, viscosity of 2%, 250 cps) was dissolved in distilled water under constant stirring at concentration of 2% w/v. Extract solution was dispersed into alginate 2% aqueous solution at drug-polymer ratio 1:2. Final suspension was spray-dried in a LM-MSD 1.0 Mini Spray-dryer with a 0.7 mm nozzle, under conditions: inlet air temperature 100 °C, outlet air temperature 50–60 °C and spray flow rate 1.0 L/h.

### 3.3. Chitosan Microparticles Preparation

For chitosan microparticles, chitosan (Sigma-Aldrich, low molecular weight, viscosity 20,000 cps) was dissolved in acetic acid 1% at concentration of 2% w/v under stirring. Extract solution was dispersed into chitosan 2% acid solution at drug-polymer ratio 1:2. The atomization conditions were the same above. Morphology and size were analysed by scanning electron microscopy (SEM-Shimadzu SS-550).

### 3.4. Microparticles Characterization

#### 3.4.1. Scanning Electron Microscopy

For size and morphology analyses, microparticles were fixed on stubs, coated with gold and observed in a Shimadzu SS-550 scanning electron microscope. Mean diameter was calculated from measurements of diameters of microparticles.

#### 3.4.2. Determination of Extract Content on Microparticles

Amount of extract in the microparticles was analysed by reversed-phase HPLC at room temperature. Microparticles containing extract were suspended in mobile phase, sonicated and filtered through a 0.45 µm pore Millipore cellulose membrane before application. Twenty µL were injected on Shimadzu L6AD Liquid Chromatograph system equipped with LC-column Shimadzu Shim-pack CLC-ODS (15 cm, 4.6 mm, 5 µm), UV/VIS detector SPD-20AV, degasser DGU-20A3. Mobile phase was 20% methanol in aqueous solution of trifluoroacetic acid 1%. Isocratic elution, wavelength 254 nm, flow rate 1.0 mL/minute and run time 20 min. A calibration curve was previously done with pomegranate extract solution in concentrations ranging from 2000 µg/mL to 62.5 µg/mL. Chromatogram showed 2 major peaks which were used as markers to quantify the extract loaded into microparticles, R^2^ = 0,998.

#### 3.4.3. *In Vitro* Extract Release Test

*In vitro* release test was carried out to analyse the extract release behavior from microparticles comparing with extract (powder). The test used USP apparatus 4. Microparticles containing 10 mg of extract and 10 mg of extract as free powder were used. The extract content was analysed by Shimadzu L6AD Liquid Chromatograph HPLC system under same conditions as cited before.

### 3.5. Antifungal *in Vitro* Assay

The minimal inhibitory concentrations (MIC) and minimal fungicidal concentrations (MFC) of pomegranate extract against *C. albicans* ATCC 10231, *C. tropicalis* ATCC 28707 and *C. glabrata* ATCC 22019 were determined according to the M27-A2 broth microdilution reference procedure of the CLSI [29] at a final inoculum of 0.5 × 10^3^ to 2.5 × 10^3^ CFU/mL, using RPMI 1640 medium with L-glutamine without bicarbonate buffered with 0.165 M MOPS (morpholine propanesulfonic acid). Extract-loaded microparticles were tested against *C. albicans*. Serial two-fold dilutions of the crude extract and microparticles were done in a microdilution plate (96 wells) containing 100 µL of sterile RPMI. Next, the inoculum was added to each well. The microplates were incubated at 37 °C for 48 h and the MIC was defined as the lowest concentration which resulted in inhibition of visual growth. 10 µL aliquots from wells with no visual growth were plated on Sabouraud Agar and incubated at 37 °C for 24 h for MFC determination. Each experiment was carried out in triplicate. Results are from three independents experiments.

### 3.6. *In Vitro* Biofilm Assay

*C. albicans* biofilm was formed on polystirene 96-well microtitre plates. Assays were done twice in triplicate. One hundred µL of a suspension containing 1 × 10^6^ cells/mL in RPMI 1640 medium with L-glutamine without bicarbonate buffered with 0.165 M MOPS were seeded in wells and incubated at 37 °C for 48 h. Well content was discharged, wells were washed with phosphate buffered saline (PBS) and several dilutions of extract of pomegranate and antifungals solutions ranging from 62.5 µg/mL to 1,000 µg/mL were added to each well. After incubation at 37 °C for 24 h, wells were rinsed with PBS. Crystal Violet (CV) staining and MTT reduction assay were carried out to evaluate the viability of biofilms. Two independent assays were carried out in triplicate.

For CV staining, 110 µL of 0.4% violet crystal solution were added to each well and incubated at room temperature for 45 min, after which plates were washed three times with sterilized distilled water to remove unadsorbed stain. 200 µL of ethanol 95% were added to each well to destaining. 100 µL of destaining solution were transferred to another plate and absorbance determined at 595 nm using a microplate reading [28].

For MTT reduction assay, slight modifications on method utilized by Schillaci *et al.* [30] were done. 50 µL of MTT solution (5 mg/mLin PBS) was added to each well and plates were incubated at 37 °C for 4 h. After staining, MTT solution was removed from each well and 100 µL of DMSO was added to dissolve MTT formazan product. 100 µL of DMSO was transferred to a new plate and optical density was measured at 570 nm using a microplate reading.

### 3.7. Cytotoxicity

Confluent Vero cell monolayers grown in 96-well cell-culture plates were incubated with a ten-fold serial dilution of extract, alginate microparticles containing extract and chitosan microparticles containing extract, starting with a concentration of 1,000 µg/mL - for 48 h at 37 °C and 5% CO_2_. At that time, cultures fixed with 10% trichloroacetic acid for 1 h at 4 °C were stained for 30 min with 0.4% Sulforhodamine B (SRB) in 1% acetic acid and subsequently washed with distilled water. Bound SRB was solubilized with 150 µL 10 mM Tris-base solution. Absorbance was read in an ELISA plate reader at 530 nm. The cytotoxicity was expressed as a percentage of the optical density compared to the control of Vero cells not treated. Each experiment was carried out in triplicate. Results are expressed as means of three independents assays.

## 4. Conclusions

Microencapsulation protects the encapsulated drug, and allows a controlled release to achieve the desired therapeutic effect. Microparticles could be incorporated in dosage forms for administration. Spray-dried microparticles containing pomegranate extract are easily prepared using either alginate or chitosan as encapsulating agents. In this study, these agents did not differ in yield, size, efficiency of encapsulation, or antifungal activity. The amount of the extract in the microparticles was quantified by HPLC, and the process of encapsulation preserved the antifungal activity of the extract against *C. albicans*, which is responsible for mycosis in the buccal region. *In vivo* studies are necessary, but these results should prove useful for developing a formulation to treat oral infections caused by *C. albicans*.
